# Supervised learning methods in modeling of CD4+ T cell heterogeneity

**DOI:** 10.1186/s13040-015-0060-6

**Published:** 2015-09-04

**Authors:** Pinyi Lu, Vida Abedi, Yongguo Mei, Raquel Hontecillas, Stefan Hoops, Adria Carbo, Josep Bassaganya-Riera

**Affiliations:** 1 The Center for Modeling Immunity to Enteric Pathogens, Virginia Bioinformatics Institute, Virginia Tech, Blacksburg, VA 24061 USA; 2 Nutritional Immunology and Molecular Medicine Laboratory, Virginia Bioinformatics Institute, Virginia Tech, Blacksburg, VA 24061 USA; 3 BioTherapeutics Inc, 1800 Kraft Drive, Suite 200, Blacksburg, VA 24060 USA

## Abstract

**Background:**

Modeling of the immune system – a highly non-linear and complex system – requires practical and efficient data analytic approaches. The immune system is composed of heterogeneous cell populations and hundreds of cell types, such as neutrophils, eosinophils, macrophages, dendritic cells, T cells, and B cells. Each cell type is highly diverse and can be further differentiated into subsets with unique and overlapping functions. For example, CD4+ T cells can be differentiated into Th1, Th2, Th17, Th9, Th22, Treg, Tfh, as well as Tr1. Each subset plays different roles in the immune system. To study molecular mechanisms of cell differentiation, computational systems biology approaches can be used to represent these processes; however, the latter often requires building complex intracellular signaling models with a large number of equations to accurately represent intracellular pathways and biochemical reactions. Furthermore, studying the immune system entails integration of complex processes which occur at different time and space scales.

**Methods:**

This study presents and compares four supervised learning methods for modeling CD4+ T cell differentiation: Artificial Neural Networks (ANN), Random Forest (RF), Support Vector Machines (SVM), and Linear Regression (LR). Application of supervised learning methods could reduce the complexity of Ordinary Differential Equations (ODEs)-based intracellular models by only focusing on the input and output cytokine concentrations. In addition, this modeling framework can be efficiently integrated into multiscale models.

**Results:**

Our results demonstrate that ANN and RF outperform the other two methods. Furthermore, ANN and RF have comparable performance when applied to *in silico* data with and without added noise. The trained models were also able to reproduce dynamic behavior when applied to experimental data; in four out of five cases, model predictions based on ANN and RF correctly predicted the outcome of the system. Finally, the running time of different methods was compared, which confirms that ANN is considerably faster than RF.

**Conclusions:**

Using machine learning as opposed to ODE-based method reduces the computational complexity of the system and allows one to gain a deeper understanding of the complex interplay between the different related entities.

## Background

### Immune cell differentiation and modeling

The process of immune cell differentiation plays a central role in orchestrating immune responses. This process is based on the differentiation of naïve immune cells that, upon activation of their transcriptional machinery through a variety of signaling cascades, become phenotypically and functionally different entities capable of responding to a wide range of viruses, bacteria, parasites, or cancer cells. Functionally, immune cells have been classified in either regulatory or effector cell subsets. The cell differentiation process involves a series of sequential and complex biochemical reactions within the intracellular compartment of each cell. The Systems Biology Markup Language (SBML) is an XML-based format widely used to represent as well as store models of biological processes. SBML allows the encoding of biological process including their dynamics. This information can be unambiguously converted into a system of Ordinary Differential Equations (ODEs). Of note, ODE models are extensively used to model biological processes such as cell differentiation, immune responses towards specific pathogens, autoimmune processes or intracellular activation of specific cellular pathways [1–3]. Several equations are usually required to adequately represent these complex immunological processes, being either at the level of the whole organism, tissue, cells or molecules

In one of our previous studies, Carbo et. al. published the first comprehensive ODE model of CD4+ T cell differentiation that encompassed both effector T helper (Th1, Th2, Th17) and regulatory Treg cell phenotypes [3]. CD4+ T cells play an important role in regulating adaptive immune functions as well as orchestrating other subsets to maintain homeostasis [4]. These cells interact with other immune cells by releasing cytokines that could further promote, suppress or regulate immune responses. CD4+ T cells are essential in B cell antibody class switching, in the activation and growth of CD8+ cytotoxic T cells, and in maximizing bactericidal activity of phagocytes such as macrophages. Mature T helper cells express the surface protein CD4, for which this subset is referred as CD4+ T cells. Upon antigen presentation, naïve CD4+ T cells become activated and undergo a differentiation process controlled by the cytokine milieu in the tissue environment. The cytokine environmental composition therefore represents a critical factor in CD4+ T cell differentiation. As an example, a naïve CD4+ T cell in an environment rich in IFNγ or IL-12 will differentiate into Th1. In contrast, an environment rich in IL-4 will induce a Th2 phenotype. Some other phenotypes are also balanced by each other: Th17 cells, induced by IL-6, IL-1β and TGF-β, are closely balanced by regulatory T cells (induced by TGFβ only) [5]. Furthermore, competition for cytokines by competing clones of CD4+ T cells within an expanding cell population (proliferation), cell death and expression of other selective activation factors such as the T cell receptor, OX40, CD28, ICOS and PD1 are key steps that influence CD4+ T cell differentiation.

Computational approaches allow concurrent multiparametric analysis of biological processes. Computational algorithms and models have become powerful and widely used tools to improve the efficiency and reduce cost of the knowledge discovery process. Systems modeling approaches combined with experimental immunology studies can integrate existing knowledge and provide novel insights on rising trends and behaviors in biological processes such as CD4+ T cell differentiation and function. The CD4+ T cell differentiation model was built upon the current paradigms of molecular interactions that occur in CD4+ T cells, which consists of 60 ODEs, 53 reactions, and 94 species. The mathematical model ensures proper modulation of intracellular pathways and cell phenotypes via external cytokines representing the cytokine milieu. Two types of kinetic equations were employed to mathematically compute dynamic biological processes in the CD4+ T cell model: 1) mass action and 2) Hill equation kinetics. Despite their simplicity, mass action kinetics are widely accepted and extensively validated in biological systems due to their inherent ability to accurately represent elementary reactions and species degradation [6]. Mass action rates are also extremely reliable for stochastic modeling simulations. In the CD4+ T cell model, the natural loss of model species due to mRNA and protein decay was fit using mass action rate laws. On the other hand, sigmoidal Hill equations were used to model more complex molecular processes that behave via “on/off” switch mechanisms including protein phosphorylation, cytokine-receptor binding and transcription. Extensive studies have demonstrated the benefits of the Hill equation for studying combinatorial regulation, especially in sigmoidal Hill equations [7], and thus this equation set captures complexities arising when a particular model species can be modified by more than one input. Results from modeling the pleiotropic and highly dynamic regulation of CD4+ T cell differentiation has guided experimentation to elucidate underlying regulatory mechanisms, identify novel putative CD4+ T cell subsets or potential targets, and enrich our understanding of the dynamics of the process [8, 9].

ODE-based modeling approaches require detailed knowledge about kinetic parameters, some of which can be estimated from literature and some from *in silico* experiments. However, models that are based on a large parameter set will be subject to higher level of inaccuracies. Thus, the use of novel modeling approaches applicable to the immune system and specifically to the CD4+ cell differentiation has a high value for investigation.

### Multiscale modeling and model reduction

**Fig. 1:**
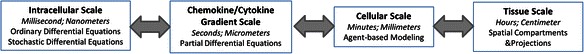
Integration of 4 order spatiotemporal scales. To be able to model fine-grained intracellular behaviors, a multiscale modeling approach which embeds intra-cellular models into the inter-cellular tissue level models is needed. The multiscale modeling approach includes four scales: Intracellular, Chemokine/Cytokine diffusion (intercellular), Cellular, and Tissue

Current biomedical research involves performing experiments and developing hypotheses that link different scales of biological systems such as: intracellular signaling or transcriptional interactions, cellular behavior and cell population behavior, as well as tissue and organism-level events. Computational modeling efforts exploring multiscale systems have to incorporate an array of techniques due to the different time and space scales involved. In one of our previous studies, Mei et. al. presented Enteric Immunity Simulator (ENISI), an agent-based simulator for modeling mucosa immune responses to enteric pathogens [10]. ENISI uses a rule-based approach and can simulate cells, cytokines, cell movement and cell-cell interactions. To be able to model fine-grained intracellular behaviors, a multiscale modeling approach that embeds intracellular models into the intercellular tissue level models is needed. Indeed, the multiscale modeling approach includes four scales: Intracellular, Chemokine/Cytokine diffusion (intercellular), Cellular, and Tissue. The current version of ENISI incorporates Cellular Scale, Chemokine Scale and Tissue Scale. The cellular scale represents how the cells interact with nearby cells and incorporates the plasticity of a cell based on stochastic and temporal rules. The chemokine scale represents the chemokine concentration and diffusion process. Finally, the tissue scale represents the spatial and compartmental information (Fig. 1).

Fine-grained ODE models of intracellular pathways controlling immune cell differentiation are adequate for studying mechanisms of cell differentiation. However, they can be highly complex and expensive from a computational stand-point, especially when embedded within large-scale agent-based simulations. ENISI Visual models a large number of cells and microbes in the gastrointestinal mucosa. If each agent is represented by 60 ODEs, as an example, the simulation will be hardly scalable. Therefore, to be able to develop efficient agent-based multiscale models, model reduction needs to be performed. In addition, multiscale models usually do not require all the internal details of intracellular scales to have predictive value. In essence, novel model reduction strategies could be used to address the multiscale scalability requirements to reduce molecular models before integrating them into large-scale agent-based tissue-level models.

### Supervised learning methods and their applications

Supervised machine learning methods use training data to learn the structure of a system and utilize that knowledge to predict the outcome for an unseen condition. Supervised learning methods have been applied in multiple areas, such as bioinformatics, cheminformatics, database marketing, spam detection, and pattern recognition in general [11]. Artificial Neural Network (ANN), Linear Regression (LR), Support Vector Machines (SVM) and Random Forest (RF) are examples of supervised machine learning methods.

Artificial neural networks algorithms, inspired by the biological neural systems, are powerful in modeling and data mining tools based upon the theory of connectionism [12]. In biological systems, neurons are connected to each other through synapses. A neuron receives inputs from multiple neurons and outputs a value based upon the activation function. Perceptron is one of the easiest data structures for the study of neural networking. The perceptron models neuron’s behavior in the following way: First the perceptron receives several input values. The connection for each of the inputs has a weight in the range of 0 to 1. The threshold unit then sums the inputs, and if the sum exceeds the threshold value a signal is sent to the output node, otherwise no signal is sent. The perceptron can learn by adjusting the weights to approach the desired output [13].

**Fig. 2:**
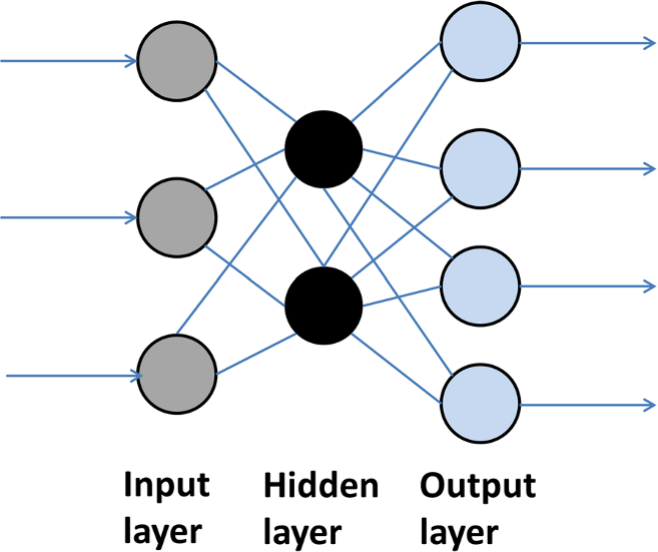
The multilayer perceptron structure of artificial neural network. The multilayer perceptron structure of artificial neural network is divided into three layers: the input layer, the hidden layer and the output layer, where each layer in this order processes inputs and deliver outputs to next layer. The extra layers give the structure needed to recognize non-linearly separable classes

Building on the algorithm of the simple perceptron, the multilayer perceptron (MLP) model not only gives a perceptron structure for representing more than two classes, it also defines a learning rule for this kind of networks. The MLP is divided into three layers: the input layer, the hidden layer and the output layer, where each layer in this order processes inputs and deliver outputs to next layer [13]. The extra layers give the structure needed to recognize non-linearly separable classes (Fig. 2). The network structures and the parameters of the activation function are important factors when developing neural network models. Feedforward neural networks are frequently used structures in modeling. There are effective learning algorithms for the parameters once the structures are set in the feedforward ANNs.

Artificial neural network algorithms are widely used for data mining tasks such as classification and pattern recognition. Neural network algorithms are especially effective in modeling non-linear relationships which makes them ideal candidates for differentiation processes. Importantly, this process is scalable. However, there are also some practical challenges. It is not possible to know in advance the ideal network topology. Therefore, ANN-based methods require testing several network settings or topologies in order to find the best solution. This technical challenge triggers an extended training period. Our initial pilot study was the first to apply neural network algorithms into studying the immune cell differentiation [14]. Based on the initial success, the study was systematized and expanded.

Linear regression model are attractive because of their simplicity and reduced computational complexity. Linear regression is an approach for modeling the relationship between a scalar dependent variable and explanatory variables [15]. In linear regression, data are modeled using linear predictor functions, while unknown model parameters are estimated from the data. Such models are called linear models [16]. Linear regression is a regression analysis that is studied rigorously, and used widely in practical applications. Linear regression is extensively used in biological [17], behavioral and social sciences to describe possible relationships between variables.

Support Vector Machines is another widely-used supervised learning algorithm for classification and regression problems. SVM contains all the main features that characterize maximum margin algorithm: a non-linear function is leaned by linear learning machine mapping into high dimensional kernel induced feature space [18]. Given a set of training examples with each marked as belonging to one category, an SVM training algorithm builds a model that could assign new examples into one category [19]. SVMs are helpful in text and hypertext categorization as their application can significantly reduce the need for labeled training instances in both the standard inductive and transductive settings. SVMs are also useful in medical science to predict survival in breast cancer [20].

**Fig. 3:**
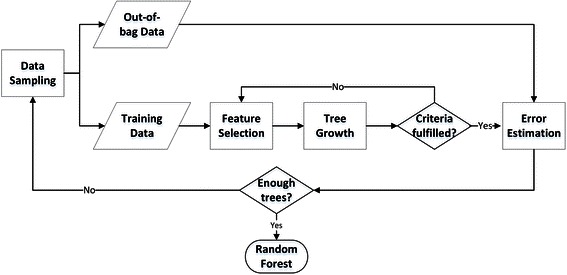
Random Forest Algorithm. Random Forest method is based on the aggregation of a large number of decision trees. Specifically, it is an ensemble of trees constructed from a training dataset and internally validated to yield a prediction of the response given the predictors for future observations

Finally bagging of classification trees is one well-known ensemble learning method. In bagging, each successive tree is independently constructed using a bootstrap sample of the dataset. A simple majority vote is then taken for prediction [21]. Based on bagging theory, Breiman proposed the Random Forest algorithm, which adds an additional layer of randomness to bagging [22]. In addition to constructing each tree using a different bootstrap data sample, random forests change how the classification and regression trees are constructed. In standard trees, each node is split using the best split among all variables, while in a random forest, each node is split using the best among a subset of predictors randomly chosen at that node. In addition, RF algorithm has only two parameters (the number of variables in the random subset at each node and the number of trees in the forest) and is usually not very sensitive to their values [23]. RF method is based on the aggregation of a large number of decision trees. Specifically, it is an ensemble of trees constructed from a training dataset and internally validated to yield a prediction of the response given the predictors for future observations. An important feature of RF is its out-of-bag (OOB) error [24]. Each observation is an OOB observation for some of the trees. The OOB error of the RF is the average error frequency obtained when the observations from the dataset are predicted using the trees for which they are OOB. Through this internal validation, the error estimation is less optimistic and usually considered as a good estimator of the expected error for independent data (Fig. 3). For instance, Random Forest models have been successfully used in recent years to explore metabolic syndrome serum profiling [25] and predict avian influenza H5N1 outbreaks [26]. However, to the best of our knowledge, this study is the first to apply RF algorithms into studying the immune cell differentiation.

### Related work

Modeling the CD4+ T cell differentiation is challenging because of the complexity of the immune system, plasticity between phenotypes, feedback loops involved in regulation and combinatorial effects of cytokines. The immune system protects the human body from pathogens by recognizing, containing, and destroying non-self or foreign antigens [27, 28]. At the highest level, the immune system can be divided into innate and adaptive branches. The innate immune system, involving cells such as macrophages, epithelial cells, neutrophils, and dendritic cells, responds quickly but non-specifically to stimuli [29]. On the contrary, the adaptive immune system involving T cells and B cells responds more specifically to antigens [30]. Immune cells are activated and differentiated into ever-growing numbers of cell subsets such as CD4+ T cells and macrophages [31–33]. These cells are regulated by different cytokines in their microenvironment. Using CD4+ T cells as an example, Th1 cells stably express IFNγ, whereas Th2 cells express IL-4. The discovery and investigation of two other CD4+ T cell subsets, induced regulatory T (iTreg) cells and Th17 cells, has led to a rethinking of the notion that helper T cell subsets represent irreversibly differentiated endpoints. Mounting evidence supports the tissue environment-dependent plasticity of CD4+ T cell subsets and suggests the emergence of new phenotypes. When both TGFβ and IL-6 are present in the environment, naïve CD4+ T cells differentiate into Th17 [34, 35]. When TGFβ alone presents in the environment, CD4+ T Cells differentiate into Treg [14]. When IFNγ and IL-12 are present, T cells differentiates into Th1 [36].

Systems biology has become an important paradigm in immunology research, using mathematical and computational models to synthesize and mine exiting knowledge, and discover new knowledge from big data [37]. Biological systems and processes can be modeled using a variety of methods [38–40]. In some instances, biological processes can be mapped to networks where nodes and edges represent biological agents such as cells and their interactions [41]. Furthermore, mathematical or computational dynamics can be applied to the network models so that *in silico* simulations can be performed [1, 42]. SBML is a XML-based file format used to represent computational models of biological processes [43]. There are many types of models used for modeling biological processes such as Bayesian networks, ODE, and agent-based models [44]. For metabolic and signaling networks, the biochemical reactions can be represented by first-order ODEs [45].

**Fig. 4:**
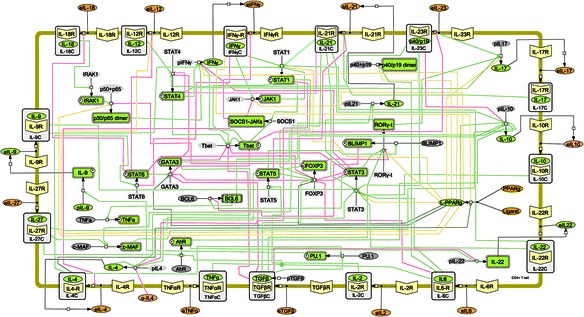
The network model of the T Cell differentiations. The figure illustrates network topologies associated with the naïve T cells differentiation towards T helper (Th)1, Th2, Th17, and induced regulatory T cells. The network is built in Systems Biology Markup Language-compliant format

In line with our systems and translational immunology efforts under Modeling Immunity to Enteric Pathogens (www.modelingimmunity.org) of computational model building, calibration, refinement and validation, Carbo et. al. published the first ODE model of CD4+ T Cell differentiation, which comprises of 60 ODEs [2]. The model as shown in Fig. 4 represents the intracellular pathways that are critical for CD4+ T cell differentiation. The hypotheses generated by this model were fully validated using *in vivo* animal models of inflammatory bowel disease (IBD). Computational modeling and mouse adoptive transfer studies were combined to gain a better mechanistic understanding of the modulation of CD4+ T cell differentiation and plasticity at the intestinal mucosa of mice. Sensitivity analyses highlighted the importance of PPARγ in the regulation of Th17 to iTreg plasticity. Indeed, validation experiments demonstrated that PPARγ is required for the plasticity of Th17 promoting a functional shift towards an iTreg phenotype. More specifically, PPARγ activation is associated with up-regulation of FOXP3 and suppression of IL-17A and RORγt expression in colonic lamina propria CD4+ T cells. Conversely, the loss of PPARγ in T cells results in colonic immunopathology driven by Th17 cells in adoptive transfer studies.

In another study Mei et. al. presented ENISI Visual, an agent-based simulator for modeling enteric immunity [10]. ENISI Visual provides high quality visualizations for simulating gut immunity to enteric pathogens and is capable of simulating gut immunity, including pathogen invasion, pro-inflammatory immune responses, pathogen elimination, regulatory immune responses, and restoring homeostasis. ENISI Visual can also help immunologists test novel hypotheses and design biological experiments accordingly. Undoubtedly a holistic model of the immune response could provide even more valuable insights; however, it needs to take into consideration complexities at the different layers: intracellular, cellular, inter-cellular, tissue and whole organism. Modeling a complex system at four levels of magnitude – multiscale modeling (MSM) – poses new set of challenges. Multiscale modeling requires considering different spatial and temporal scales, ranging from nano-meters to meters and nano-seconds to years. Therefore, different technologies have to be integrated to provide most accurate predictions. Multiscale modeling frameworks have been recently developed and attempted to address some of these challenges [46–48]. In our recent work, we have developed ENISI MSM, a multiscale modeling platform driven by high-performance computing and designed specifically for computational immunology, which integrates agent based modeling (ABM), ODEs and partial differential equations (PDEs) [48]. Our ENISI MSM platform is calibrated with experimental data and tested for the CD4+ T cell differentiation model which is able to perform a variety of *in silico* experimentation for generating new hypothesis. However, running simulations on the MSM platform that requires COPASI [49] to solve complex ODEs is computationally expensive and time consuming. Replacing the ODE-based steps in the MSM by machine learning methods would significantly improve its computational performance and allow researchers to perform broad and comprehensive *in silico* experimentation that uncovers emerging properties of the immune system and results in new nonintuitive computational hypotheses about immune responses.

Machine learning methods – supervised learning methods in particular – are key in building predictive models from observations, therefore facilitating knowledge discovery for complex systems. Neural network algorithm is a supervised learning approach and has been widely used in data mining tasks [50, 51] as well as medical applications [52, 53]. Snow et al. developed neural networks for prostate cancer diagnosis and prognosis [54]. Lek et al. introduced neural networks in ecological modeling [55]. Brusic et al. used neural networks for predicting major histocompatibility complex (MHC) binding peptides [56]. Learning is an important research topic in neural networks. White presented neural network learning algorithms from the statistical perspective [57]. Hagan et al. presented an effective learning algorithm called back-propagation for training feedforward networks [58]. In addition to modeling and predictions, neural network algorithm has also been used for solving ordinary and partial differential equations [59].

Our initial work [14] presented ANN as an alternative to solving ODEs using *in silico* data; in that study ANN was compared with LR model and it was shown to outperform the latter. In the present work, we compare four different learning methods: ANN, LR, SVM and RF. We optimize the parameters of the models and apply them to *in silico* data with and without added noise. We corroborate our findings with experimental data and demonstrate that both ANN and RF are capable of predicting the dynamic behavior of the output cytokines in four out of five cases. Finally, we also evaluated the methods based on their computational performance.

## Methods

To model cell differentiation we first define the problem and make the following assumptions. There are *m* input cytokines that regulate immune cell differentiation: C_i1_, C_i2_, …, C_im_. There are also *n* output cytokines secreted by immune cells: C_o1_, C_o2_, …, C_on_. The cytokine concentrations are positive continuous values.

The problem of modeling immune cell differentiation is to develop one model for the following functional relationship:

1 $$ \left\{{\mathrm{C}}_{\mathrm{o}1},{\mathrm{C}}_{\mathrm{o}2}, \dots,\ {\mathrm{C}}_{\mathrm{o}\mathrm{n}}\right\}={\mathrm{F}}_{\mathrm{c}}\left({\mathrm{C}}_{\mathrm{i}1},{\mathrm{C}}_{\mathrm{i}2}, \dots,\ {\mathrm{C}}_{\mathrm{i}\mathrm{m}}\right) $$

The model is designed to predict the output cytokine concentrations given concentrations of input cytokines.

### T cell differentiation process as a use case

This study focuses on the T cell differentiation. However, the techniques and algorithms developed herein can be applied to differentiations of other types of immune cells, such as macrophages, dendritic cells, B cells, etc. The input cytokines are internalized by the naïve T cells and regulate the T cell differentiation process. The output cytokines are externalized and secreted.

### Data for training and testing models

The data for modeling the relationship from the input and output cytokines can be derived from the T Cell differentiation ODE model [2] which was calibrated using data from biological experiments. By changing the concentrations of the input cytokines, the steady state of the ODE model is calculated. The steady state results provide a measure of the output cytokines that can be used in the model. Creation of datasets was achieved by using the parameter scan task of COPASI tool [49]. COPASI is a software application for simulation and analysis of biochemical networks and their dynamics, which supports models in the SBML standard and can simulate their behavior using ODEs. A five-dimensional scan was performed, where five output cytokines were independently measured. All the data is normalized to the range of [0, 1]. The method used to create datasets is equal-distance sampling. For each input cytokine, five values were chosen (0, 0.25, 0.5, 0.75, and 1). Since there is a total of five input cytokines, 625 data points were created by the parameter scan process. One hundred of the data points were selected randomly for training and the remaining 525 data points were used for testing. Additionally, uniformly distributed noise was added to the output for a quantitative analysis. Table 1 shows an example of data points used in the study.

**Table 1:** Example datasets used for training and testing the models

Sample data	Input data				Output data				
	IFNγ	IL12	IL6	TGFβ	IL17	RORgt	IFNγ	Tbet	FOXP3
Data without Noise	1	0	0.5	0	0.996	0.989	0.122	0.547	7.51E-06
	0.75	0.75	0	0.75	0.156	0.117	0.942	0.677	0.000103
	0.5	0.5	0.25	0.5	0.989	0.967	0.282	0.404	1.25E-05
	0.25	0	0	1	0.155	0.117	0.401	0.645	0.000105
Data with noise in range of [−0.5 %, 0.5 %]	1	0	0.5	0	0.974	0.913	0.118	0.545	7.12E-06
	0.75	0.75	0	0.75	0.148	0.106	0.900	0.644	9.91E-05
	0.5	0.5	0.25	0.5	0.950	0.922	0.264	0.391	1.28E-05
	0.25	0	0	1	0.144	0.115	0.390	0.640	0.000105
Data with noise in range of [−1 %, 1 %]	1	0	0.5	0	0.933	0.880	0.114	0.482	6.71E-06
	0.75	0.75	0	0.75	0.133	0.116	0.784	0.614	9.84E-05
	0.5	0.5	0.25	0.5	0.980	0.959	0.264	0.368	1.21E-05
	0.25	0	0	1	0.154	0.106	0.352	0.604	9.75E-05

### Supervised learning methods

#### Artificial neural networks

**Fig. 5:**
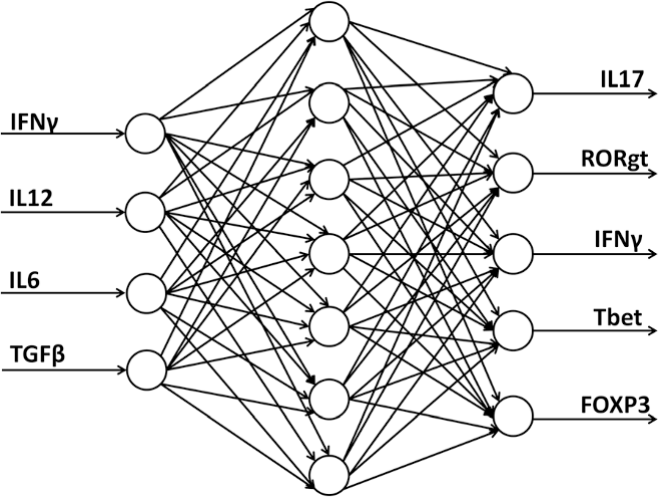
Artificial neural network (ANN) model of CD4+ T cell differentiation. The ANN model for T cell differentiation was built using a package in R named neuralnet. The network of four inputs and five outputs, seven hidden neurons were necessary to best model the complex non-linear system of cell differentiation using back-propagation

ANN models can be used to model nonlinear relationships. We developed the ANN model for T cell differentiation using a package in R named neuralnet [60]. The learning algorithm used is back-propagation. The function neuralnet is used for training neural networks, which provides the opportunity to define the required number of hidden layers and hidden neurons. The most important arguments of neuralnet function include formula (a symbolic description of the model to be fitted), data (a data frame containing the variables specified in formula), and a hidden vector (specifying the number of hidden layers and hidden neurons in each layer) [60]. To optimize the performance of the ANN model, we tested different sizes of hidden layers, including 1, 2, 4, 5, 6, 7, 8, 10, and 11 hidden neurons. By comparing the average absolute difference between the model predictions and real outputs from the test data, the neural network model with seven hidden neurons was identified to perform best (Table 2). Size of hidden layers is a critical model parameter. If the number of layers is too small under-learning can occur whereas a size too large can cause over-learning or over fitting. In this study, our results demonstrated that with the network of four inputs and five outputs, seven hidden neurons were necessary to best model the complex non-linear system of cell differentiation using back-propagation (Fig. 5).

**Table 2:** Prediction errors of the neural network models with different sizes of hidden layer

Number of hidden neurons	IL17	RORgt	IFNγ	Tbet	FOXP3	Sum of prediction error
1	0.0551	0.0408	0.0831	0.114	0.0233	0.316
2	0.0559	0.0415	0.049	0.114	0.0369	0.297
4	0.0562	0.0411	0.0527	0.109	0.0362	0.295
5	0.0562	0.0415	0.0367	0.0396	0.0368	0.211
6	0.0562	0.0423	0.0482	0.0436	0.0357	0.226
7	0.0561	0.0419	0.0407	0.0142	0.0368	0.190
8	0.0561	0.0421	0.0426	0.0234	0.0368	0.201
10	0.0561	0.0415	0.0503	0.0453	0.0362	0.230
11	0.0561	0.0424	0.0423	0.0148	0.0360	0.192

#### Linear regression

LR model was tested for its simplicity. R has a linear regression module lm that was adapted and used in this study. The lm function is used to fit linear models, which can be used to carry out regression, single stratum analysis of variance and analysis of covariance [61].

#### Support vector machine

A model was created using SVM algorithm. The R Package, e1071 [62], was applied to build the support vector machine models using the same training data and test data as used by our previous modeling approaches. To optimize the performance of the SVM model, we tested different width of radial kernel, including baseline (0.25), 1, 0.1, 0.01, and 0.001. By comparing the average absolute difference between the model predictions and real outputs from the test data, the model with a kernel size of 0.25 (baseline) was identified to perform best (Table 3).

**Table 3:** Prediction error of support vector machine models with different width of radial kernel. The baseline width is the inverse of the dimension of the data (in this case Baseline will be 0.25)

Width of radial	IL17	RORgt	IFNγ	Tbet	FOXP3	Sum of prediction error
Baseline	0.181	0.179	0.146	0.122	0.0355	0.665
1	0.189	0.192	0.149	0.126	0.0349	0.691
0.1	0.193	0.192	0.160	0.130	0.0360	0.711
0.01	0.257	0.263	0.216	0.148	0.0366	0.920
0.001	0.343	0.351	0.259	0.174	0.0368	1.163

#### Random forest

**Fig. 6:**
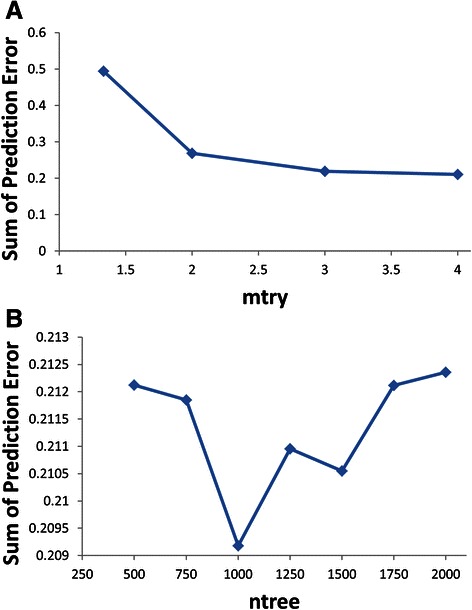
Performance optimization of Random Forest (RF) model. The RF model was created using the randomForest package in R. To optimize the performance of the RF model, two main variables – *mtry* (numbers of variables randomly sampled as candidates at each split) and *ntree* (numbers of trees to grow) – were optimized. The RF model with 1000 trees and 4 variables randomly sampled as candidates at each split was identified to perform best

A RF model was created using the randomForest package in R [23]. The function randomForest is used for building trees, which provides the opportunity to define the number of trees to grow and the number of variables randomly sampled as candidates at each split. For each output cytokine, a Random Forest model was built. In essence, for five outputs, IL17, RORgt INFγ, Tbet, and FOXP3, five Random Forest models were created. To optimize the performance of the RF model, two main variables – *mtry* and *ntree* – were optimized (see Fig. 6). By comparing the average absolute difference between the model predictions and real outputs from the test data, the random forest model with 1000 trees and 4 variables randomly sampled as candidates at each split was identified to perform best.

### Capability of the models to analyze data with noise

Stochasticity is an inherent component of biological processes and an important aspect in modeling such systems [43–46]. Thus, we incorporated noise to the output data points. A uniformly distributed noise in range of [−0.5 %, 0.5 %] and [−1 %, 1 %] was add to all five output data points independently in order to assess whether the learning methods could be used to model the system with same level of accuracy. The level of noise that was applied is relatively low because there is no indication of any species in the model to be subject to a low copy number and therefore to significantly higher levels of fluctuation and noise. In a similar manner, 100 data points were selected randomly as the training dataset and the remaining 525 data were used for testing.

### Model validation

#### Testing the models using in vitro data

*In vitro* data were obtained from recent publications, which were used to further validate neural network model and random forest model. The first publication [63] shows that TGFβ and IL-6 are required for the lineage commitment of pathogenic Th17 cells. TGFβ and IL-6 drive the production of IL-17 by T cells and restrain Th17 cell-mediated pathology, such as production of IFNγ. The second publication [64] shows that TGFβ and IL-6 together induces the differentiation of pathogenic Th17 cells from naïve T cells. Meanwhile, IL-6 as an acute phase protein induced during inflammation, completely inhibits the generation of Foxp3+ Treg cells induced by TGFβ. The third publication [65] shows that T-bet upregulation and subsequent IL-12 stimulation are essential for induction of Th1 mediated immunopathology in Crohn’s disease. Also, augmentation of IFNγ production by IL-12/IL-18 was independent of T-bet expression. The experimental condition (cytokine concentrations) was extracted from publications as inputs of supervised learning models. The outputs of models were compared with experimental data to identify whether the dynamics behaviors of cells could be predicted correctly.

#### 10-fold cross validation

Cross-validation is a model validation technique for assessing how the results of a statistical analysis could generalize to an independent dataset. The goal of cross validation is to define a dataset to test the model in the training phase, in order to limit overfitting and give an insight on how the model will generalize to an independent dataset. 10-fold cross-validation approach was used in our studies. Firstly, the whole dataset was randomly divided into 10 subsets. Since there are totally 625 data samples, five subsets contains 63 data respectively and the other five subsets have 62 data per set. Then, 10 rounds of learning were performed for each machine learning approaches; on each round one subset of the data was used as a test set and the remaining 9 data subsets are used as training data. The average of mean-squared error on the predictions of each machine learning approach was used as an estimate of accuracy.

### Running time comparison among different supervised learning methods

The running time of different supervised learning methods was calculated using proc.time function of R, which determines how much real and CPU time the currently running R process has already taken.

## Results

LR model was tested for its simplicity. R has a linear regression module lm that was adapted and used in this study. The lm function is used to fit linear models, which can be used to carry out regression, single stratum analysis of variance and analysis of covariance [61]. The result of the linear regression model can be summarized as linear transformation from the input cytokines to the output cytokines as shown by the Eq. 2. The transformation matrix, M_Tran_, (Eq. 3) summarizes the relationship between input and output cytokine concentrations.

2 $$ \left[\begin{array}{c}\hfill FOXP3\hfill \\ {}\hfill IF{N}_{\gamma i}\hfill \\ {}\hfill IL17\hfill \\ {}\hfill RO{R}_{\gamma t}\hfill \\ {}\hfill Tbet\hfill \end{array}\right]={M}_{Tran} \times \left[\begin{array}{c}\hfill 1\hfill \\ {}\hfill IF{N}_{\gamma o}\hfill \\ {}\hfill IL12\hfill \\ {}\hfill IL6\hfill \\ {}\hfill TF{G}_{\beta}\hfill \end{array}\right] $$

3 $$ {M}_{Tran} = \left[\begin{array}{c}\hfill 0.0386\hfill \\ {}\hfill -0.0259\ \hfill \\ {}\hfill -0.0303\hfill \\ {}\hfill - 0.0191\hfill \\ {}\hfill 0.00558\hfill \end{array}\begin{array}{c}\hfill 0.531\hfill \\ {}\hfill \kern0.5em -0.0536\hfill \\ {}\hfill 0.297\hfill \\ {}\hfill -0.568\hfill \\ {}\hfill 0.0551\hfill \end{array}\begin{array}{c}\hfill 0.408\hfill \\ {}\hfill 0.155\hfill \\ {}\hfill \kern0.5em -0.0466\hfill \\ {}\hfill 0.773\hfill \\ {}\hfill -0.130\hfill \end{array}\begin{array}{c}\hfill 0.387\hfill \\ {}\hfill 0.146\hfill \\ {}\hfill \kern0.75em -0.0592\hfill \\ {}\hfill 0.811\hfill \\ {}\hfill -0.132\hfill \end{array}\begin{array}{c}\hfill 0.663\hfill \\ {}\hfill 0.0267\hfill \\ {}\hfill 0.129\hfill \\ {}\hfill \kern0.75em -0.302\hfill \\ {}\hfill -0.198\hfill \end{array}\right], $$

where rows represent IFNγ, IL12, IL6, and TGFβ respectively.

The prediction error, the average absolute difference between the model predictions and real outputs from the test data, of the linear model is shown in Table 3. Considering the data are normalized within [0, 1], the prediction error of linear regression model is larger than that of neural network model. This corroborates that the T cell differentiation process is highly non-linear and linear regression will not be an appropriate method for this highly complex and non-linear process. By calculating the prediction error, it is concluded that the performance of support vector machine model is better than linear regression model, but worse than neural network model (Table 4). The prediction error of the RF model (Table 4) is better than linear regression and support vector machine. RF’s performance is comparable with ANN method.

**Table 4:** Comparison of prediction error for the different models

Approach	IL17	RORgt	IFNγ	Tbet	FOXP3	Sum of prediction error
Artificial Neural Network	0.0561	0.0419	0.0407	0.0142	0.0368	0.190
Linear Regression	0.256	0.258	0.213	0.141	0.0362	0.904
Support Vector Machine	0.181	0.179	0.146	0.122	0.0355	0.665
Random Forest	0.0261	0.032	0.0326	0.0920	0.0296	0.211

The Table 5 shows that the ANN model and RF model still outperform the linear regression model and the support vector machine model when noise is added to the data. However, the performance of these two models deteriorates slightly when compared to data without added noise.

**Table 5:** Comparison of prediction error on data with noise for the different models

Noise level	Approach	IL17	RORgt	IFNγ	Tbet	FOXP3	Sum of prediction error
Uniformly distributed noise in range of [−0.5 %, 0.5 %]	Artificial Neural Network	0.0671	0.0698	0.042	0.0362	0.0354	0.250
	Linear Regression	0.235	0.235	0.190	0.129	0.0355	0.824
	Support Vector Machine	0.0329	0.146	0.182	0.178	0.111	0.649
	Random Forest	0.0413	0.0479	0.0364	0.0769	0.0397	0.242
Uniformly distributed noise in range of [−1 %, 1 %]	Artificial Neural Network	0.0706	0.0553	0.0435	0.0361	0.0393	0.2448
	Linear Regression	0.795	0.682	0.677	0.546	0.46	3.16
	Support Vector Machine	0.179	0.177	0.147	0.112	0.0406	0.6556
	Random Forest	0.0552	0.0495	0.0484	0.0935	0.0349	0.2815

Testing the best performers – ANN and RF – using *in vitro* data is important in order to assess the predictive quality of the models. We gathered three publications that provide experimental data for our input and output cytokines. We tested the methods and in four out of five cases both models were able to predict the dynamic behavior of the system. For instance in the first study, when IL-6 and TGFβ are 100 and 10 respectively the level of IL-17 is measured to be 4875 (baseline level is 188), corresponding to an up-regulation of IL-17 with respect to baseline. The model predictions for the same input values are 0.99 versus 0.14 for ANN and 0.769 versus 0.128 for RF: and up-regulation with respect to the baseline. Four out of five experimental data are reproduced with the correct dynamic behavior. It is essential to note that even though it is important to test the system with experimental data, there is discrepancy between different experimental conditions and the final results. For instance, the third and fourth studies measure the same cytokines; however, the results demonstrate different dynamic behavior. In the third study the authors observe an up-regulation of Foxp3; while, in the fourth study authors observe a down-regulation of the same output. The difference can be due to experimental conditions as well as input range (Table 6).

**Table 6:** Applying the RF and ANN method on experimental data

Study	Input			Output												Dynamics behavior	
				Experimental (EXP)				Model prediction (ANN)				Model prediction (RF)				Up/Down-regulation with respect to control	
	IL12	IL6	TGFβ	IL17	RORgt	FOXP3	Tbet	IL17	RORgt	FOXP3	Tbet	IL17	RORgt	FOXP3	Tbet	EXP	ANN / RF
1.[63]		0	0	188				0.14				0.128				↑	↑ / ↑
		100	10	4875				0.99				0.769					
2.[63]		0	0		63				0.104				0.103			↑	↑ / ↑
		100	10		485				0.999				0.946				
3.[63]		0	0			406				0.0164				0.194		↑	↓ / ↓
		100	10			469				0.0075				0.143			
4.[64]		0	0			3.3				0.0164				0.194		↓	↓ / ↓
		20	3			0.6				0.0065				0.143			
5.[65]	0						1				0.0602				0.874	↑	↑ / ↑
	10						1.33				0.809				0.992		

In order to control overfitting and give an insight on how the models will generalize to an independent dataset cross-validation was performed. Cross-validation is a model validation technique for assessing how the results of a statistical analysis would generalize to an independent dataset in a practical setting. The goal of cross-validation is to define a dataset to test the model in the training phase. 10-fold cross-validation approach was used to evaluate models created by different machine learning, including ANN, LR, SVM, and RF. The average of mean-squared error on the predictions of each model was used as an estimate of accuracy, which is shown in Table 7. ANN and RF are still the best performers following this analysis.

**Table 7:** Comparison of average prediction error on data from 10-fold cross validation for the different models

Approach	IL17	RORgt	IFNγ	Tbet	FOXP3	Sum of prediction error
Artificial Neural Network	0.00662	0.0128	0.0124	0.00755	0.0201	0.0595
Linear Regression	0.239	0.241	0.201	0.136	0.0311	0.849
Support Vector Machine	0.0914	0.0881	0.0893	0.0871	0.0277	0.384
Random Forest	0.000421	0.000647	0.00131	0.0210	0.00660	0.030

The R function, proc.time, was used to determine how much real and CPU time (in seconds) the training and testing processes of each supervised learning methods have already taken (Table 8). proc.time returns five elements for backwards compatibility, but its print method prints a named vector of length 3. The first two entries are the total user and system CPU times of the current R process and any child processes on which it has waited, and the third entry is the ‘real’ elapsed time since the process was started. The system specification is Intel® Core(TM) i7-4500 CPU @ 1.80 GHz 2.40 GHz and 4.00 GB RAM. The comparison between ANN and RF shows that ANN is faster on both real and CPU time.

**Table 8:** Running time comparison for the different models

Approach	Running time for training (s)			Running time for testing (s)		
	User	System	Elapsed	User	System	Elapsed
Artificial Neural Network	0.36	0.05	0.64	0.01	0.02	0.03
Linear Regression	0.02	0.04	0.31	0	0.02	0.02
Support Vector Machine	0.04	0.06	0.3	0	0.02	0.02
Random Forest	1.28	0.09	1.42	0.1	0.05	0.66

## Discussion

In this study, we presented four different supervised learning methods – ANN, LR, SVM, and RF – to model the CD4+ T Cell differentiation. Immune cell differentiation is an important immunological process that is not fully characterized. Based upon our previous studies on the ODE model of CD4+ T cell differentiation and agent-based modeling for enteric immunity, it is concluded that developing multiscale models requires significant reduction of the intracellular ODE model before integrating them into the inter-cellular agent-based models. However, since immune cell differentiation is a highly non-linear process, the linear regression model was not capable of fitting the data well. Linear regression models provide a simplistic approach that is very well scalable and was shown to outperform neural network models in a recent study [66]. ANN and RF were shown to be best performers with *in silico* data with and without added noise.

In particular a feed-forward neural network model has been developed, focusing on modeling the relationship between the input external cytokines regulating the cell differentiation and the output cytokines secreted and externalized by the immune cell subsets. After training using back propagation algorithm, this neural network model predicts the concentrations of the output cytokines with an average prediction error of 0.0379 for the five output cytokines concentrations. The neural network model significantly reduces the ODE model complexity by focusing on the needs of multiscale models. This approach is scalable and can be integrated into future multiscale modeling efforts.

In our analysis, we also explored SVM as potential candidates for the modeling of T cell differentiation. SVMs provide a number of advantages over ANN. For instance, ANN algorithm is more prone to over-fitting as compared to SVMs [67]. In additions, unlike ANN, computational complexities of SVM do not depend on the dimensionality of the input space [68] and therefore it could provide a more scalable framework. Finally, solution to SVM is global [69], where ANN could suffer from multiple local minima [70]. However, in our analysis the ANN significantly outperformed SVM.

Random Forest has also outperformed SVM and Linear regression model in our experiment. RF and ANN have comparative performance. Inherited from classification and regression trees, random forest algorithm has the following advantages. It handles categorical predictors, highly non-linear interactions, and missing values. It is computationally simple even for large problems. Furthermore, RF does not require formal distributional assumptions (non-parametric) and provides an automatic variable selection process [22, 71]. In addition, it also overcomes disadvantages of classification and regression trees. For example, on accuracy aspect, random forest turns out to perform very well compared to many other classifiers, including discriminant analysis, support vector machines and neural networks [23], and is robust against over-fitting [22]. Random forest methods have been widely applied in bioinformatics and computational biology. A major field of application of RF method is large-scale genetic association studies. The response is typically a phenotype of interest, while the predictors are genetic markers, often SNPs that can be seen as predictors with two or three categories. RFs yield both a prediction tool and a ranking of the SNPs with respect to their classification ability [72]. Other applications of random forest include prediction of patient outcome from high dimensional gene expression data, where patients are instances and their outcome is the response to be predicted [73]. Another class of applications is the prediction of molecule properties based on sequence information, such as the prediction of replication capacity based on HIV-1 sequence variation [74]. However, RF methods have not been heavily used in immunology studies. To the best of our knowledge this study is the first one applying the random forest algorithm for immune cell differentiation.

Furthermore, analysis of data with noise is an important step, as biological systems are stochastic processes in general. As we have shown the performance of the ANN system deteriorates but only marginally. RF outperforms all the other methods when low level noise is added to the *in silico* data, while ANN performs best using date with high level noise. Therefore, the constructed modeling framework is stable and robust to slight variations.

Finally, by testing the best performers – ANN and RF – using *in vitro* experimental data, the predictive accuracy of the models was accessed. In four out of five cases both models were able to predict the dynamic behavior of the system, which demonstrated that our models are capable of predicting dynamic behaviors of cell differentiation system with high accuracy. There are two contradictory data on FOXP3. One data showed that Foxp3 did not substantially change (slightly increase) [63], while another data showed that addition of TGFβ plus IL-6 to T cells during differentiation completely abrogated the expression of Foxp3 [64]. This contradictory observation could be due to different background of mice used in these two studies or different initial expressing level of Foxp3 in T cells [75]. In addition to prediction accuracy, the running time of each supervised learning method was measured. It is concluded that ANN performs more efficiently than RF with similar accuracy.

## Conclusions

This is the first study using neural networks as well as random forest to model immune cell differentiation. We have shown that the proposed modeling framework is robust to noise, and outperforms two other widely used methods – LRM and SVM. Furthermore, ANN and RF models represent ideal candidates for integration into the agent-based models that we have developed using ENISI MSM to study the immunological processes comprehensively and systematically. Using machine learning as opposed to ODE-based methods will reduce the computational complexity of the system and allow us to gain a deeper understanding of the complex interplay between the molecules, cells and tissues of the immune system to advance the development of safer and more efficacious therapeutics.

## Abbreviations

ANN: Artificial neural networks
ENISI: Enteric immunity simulator
iTreg: Induced regulatory T
LR: Linear regression
MHC: Major histocompatibility complex
MLP: Multi layer perceptron
MSM: Multi scale modeling
ODE: Ordinary differential equation
OOB: Out-of-bag
RF: Random forest
Treg: Regulatory T cells
SVM: Support vector machines
SBML: Systems biology markup language
Th: T helper
